# CXCL9 as a Prognostic Inflammatory Marker in Early-Stage Lung Adenocarcinoma Patients

**DOI:** 10.3389/fonc.2020.01049

**Published:** 2020-06-30

**Authors:** Yanwei Zhang, Beibei Sun, Minjuan Hu, Yuqing Lou, Jun Lu, Xueyan Zhang, Huimin Wang, Jialin Qian, Tianqing Chu, Baohui Han

**Affiliations:** ^1^Department of Pulmonary Medicine, Shanghai Chest Hospital, Shanghai Jiao Tong University, Shanghai, China; ^2^Institute for Thoracic Oncology, Shanghai Chest Hospital, Shanghai Jiao Tong University, Shanghai, China

**Keywords:** CXCL9, inflammation, early stage, lung adenocarcinoma, prognosis

## Abstract

**Background:** This study was performed to evaluate the value of inflammatory biomarkers in predicting the prognosis of early-stage (stage IA-IIB) lung adenocarcinoma.

**Methods:** Ten inflammatory biomarkers were tested with a Luminex bead-based assay in early-stage lung adenocarcinoma patients who underwent resection.

**Results:** A total of 152 early-stage lung adenocarcinoma patients were analyzed in this study. The mean patient age (SD) was 59.9 (9.4) years. In total, 58.6% of patients were females, and never smokers accounted for 84.0%. Lung adenocarcinoma patients with high CXCL9 levels had a 71% reduced risk of recurrence relative to patients with low CXCL9 levels (*HR* = 0.29, 95% CI: 0.13–0.64, *p* = 0.0021). After Bonferroni correction, CXCL9 remained significantly related to the risk of early-stage lung adenocarcinoma recurrence. Lung adenocarcinoma patients with high CXCL9 levels had an 80% reduced risk of death relative to patients with low CXCL9 levels (*HR* = 0.20, 95% CI: 0.05–0.78, *p* = 0.021), and those in the TCGA validation cohort were at a 29% reduced risk of death (*HR* = 0.71, 95% CI: 0.45–0.99, *p* = 0.044).

**Conclusion:** Our results demonstrate for the first time that the CXCL9 level is a protective factor for both disease-free survival (DFS) and overall survival (OS) in early-stage lung adenocarcinoma patients.

## Introduction

Non-small cell lung cancer (NSCLC) accounts for approximately 85% of all lung cancers and is the leading cause of death from cancer worldwide (1). Adenocarcinoma is the most frequent subtype of NSCLC, and its incidence continues to increase (2–4). Lung cancer patients diagnosed in the early stages have a good prognosis, but approximately 30–55% of patients develop recurrence despite resection (5). Therefore, the identification of biomarkers able to predict which early-stage patients have a high risk for recurrence and death in subsequent years is urgently needed.

There is a growing body of evidence showing that chronic inflammation is a hallmark of lung carcinogenesis (6–8). Some broad investigations have also proven that inflammatory markers are associated with the risk of lung cancer (9–11). One of our previous studies evaluated the relationship between 10 inflammatory markers and early-stage lung adenocarcinoma risk (12), and the results showed that four inflammatory markers were significantly associated with the risk of early-stage lung adenocarcinoma: CXCL13 (C-X-C motif chemokine ligand 13), CCL22 (C-C motif chemokine ligand 22), CXCL9 (C-X-C motif chemokine ligand 9), and IL-10 (interleukin 10). After Bonferroni correction, only CXCL13 and CCL22 were found to be independently related to the risk of early-stage lung adenocarcinoma.

However, few studies have used a broad panel to evaluate the prognostic value of inflammatory markers in early-stage lung adenocarcinoma. To fill this gap, the present study therefore aimed to investigate the association between these 10 inflammatory markers and the prognosis of early-stage lung adenocarcinoma patients with a median follow-up time of 60.6 months. The 10 inflammatory biomarkers were measured in all study participants, including CRP (C-reactive protein), CCL22, CXCL13, CXCL9, TNFRII (Tumor necrosis factor receptor II), IL-1b (interleukin 1 beta), IL-6 (interleukin 6), IL-10, IFN-r (interferon-gamma), and TGF-a (transforming growth factor alpha), and they were previously reported to be associated with lung cancer (13–18).

## Materials and Methods

### Study Population

Patients who were histologically confirmed as having lung adenocarcinoma by lung resection according to the classification criteria of the World Health Organization (19) at Shanghai Chest Hospital between September 2013 and March 2015 were included in the present study. The pathologic stage was determined according to the International Association for the Study of Lung Cancer (IASLC) tumor-node-metastasis (TNM) classification, 8th edition (20). The inclusion criteria were as follows: (1) patients with a TNM stage IA to IIB; (2) available serum samples before diagnosis; and (3) complete follow-up data. In contrast, patients were excluded if they were missing baseline data. All the enrolled patients signed informed consent documents. This study was approved by the ethics committee of Shanghai Chest Hospital.

### Follow-Up Assessment

Follow-up was performed annually after discharge at the outpatient department of our hospital or through a telephone interview or post mail if the patient failed to show at the scheduled time. The main endpoint was disease-free survival (DFS), defined as the time from lung resection to the date of disease relapse or death from any cause, whichever occurred first. Patients who did not experience any event were censored at the date of the last follow-up in April 2019. The secondary endpoint was overall survival (OS), defined as the time from surgery until the date of death or the last follow-up, whichever occurred first.

### Laboratory Methods

The circulating levels of 10 inflammatory markers were measured in serum specimens collected at baseline (processed at 2,400–3,000 rpm for 15 min), and then the samples were frozen within 2 h of collection and stored at −80°C. Inflammatory markers were measured using a Luminex bead-based assay (12). Concentrations were calculated using a four- or five-parameter standard curve. All the samples were assayed in duplicate, and the detection results were averaged for analysis.

### Statistical Analysis

Data are summarized as the mean ± standard deviation (SD) for continuous variables and as counts and percentages for categorical variables. Their differences were compared by the Wilcoxon rank-sum test, Student's *t*-test, Pearson's X2 test or Fisher's exact test, where appropriate. A survival tree analysis was employed to determine the optimal cutoff point of each biomarker as described previously (21, 22) (STREE software, available at http://c2s2.yale.edu/software/stree/).

For inflammatory biomarkers, measurements below the lowest limit of detection (LLOD) were assigned a value of half the LLOD, as described previously (12, 13). Biomarkers in which the levels were over the LLOD in more than 25% of subjects were dichotomized by the optimal cutoff points determined by the survival tree analysis. Biomarkers in which the levels were over the LLOD in 10–25% of individuals were categorized as detectable and undetectable.

The impact of each biomarker on DFS or OS in lung adenocarcinoma patients was evaluated by a multivariate Cox proportional hazards model and is as expressed as the hazard ratio (HR) and its 95% confidence interval (CI) after controlling for age, sex, smoking status, TNM stage, and neoadjuvant therapy. The Kaplan–Meier (K–M) method and log-rank test were used to compare DFS and OS between patient groups defined by each dichotomized biomarker. Stratified analyses were performed separately according to age, sex, and smoking status. The findings were validated using mRNA expression and OS data from The Cancer Genome Atlas (TCGA) for lung adenocarcinoma (www.cbioportal.org; accessed July 2019).

All statistical analyses were completed with SPSS software version 22.0 for Windows (IBM SPSS, Inc., Chicago, IL, USA) unless indicated. Statistical significance was taken as a two-sided *P* < 0.05, and multiple testing was controlled by Bonferroni correction.

## Results

### Patient Characteristics

A total of 152 early-stage lung adenocarcinoma patients were ultimately analyzed in this study, and the patient demographics are shown in Table 1. The mean patient age (SD) was 59.9 (9.4) years. Females accounted for 58.6% of the total population, and 84.0% were never smokers. According to TNM stage, 71.7% of patients were at stage I, and 28.3% of patients were at stage II. The median values of the inflammatory markers were comparable between the recurrence and non-recurrence groups (all *p* > 0.05). The optimal cutoff points for CXCL9, CXCL13, CCL22, TNFRII, IL-6, and CRP were 7.8 pg/ml, 21.4 pg/ml, 77.7 pg/ml, 275.4 pg/ml, 14.1 pg/ml, and 888244.7 pg/ml, respectively. The median follow-up time was 60.6 months.

**Table 1:** Baseline characteristics of lung adenocarcinoma patients.

**Variables**	**Non-recurrence cases**	**Recurrence cases**	***P*-value**
Age (years), mean (SD)	59.6 (9.6)	58.2 (10.7)	0.61
Gender, N (%)			
Female	70 (64.2)	19 (44.2)	0.024
Male	39 (35.8)	24 (55.8)	
Smoking status, N (%)			
Never smokers	94 (86.2)	34 (79.1)	0.28
Ever smokers	15 (13.8)	9 (20.9)	
TNM stage, N (%)			
IA	82 (75.2)	10 (23.3)	<0.001
IB	11 (10.1)	6 (14.0)	
II	16 (14.7)	27 (62.8)	
Adjuvant therapy			
Yes	21 (19.3)	13 (30.2)	0.14
No	88 (80.7)	30 (69.8)	
Biomarkers(pg/mL), mean (SD)			
CRP	2199847.0 (371273.9)	22226112 (323723.1)	0.97
CXCL13	48.7 (25.6)	45.0 (27.0)	0.43
CCL22	90.0 (48.1)	93.5 (42.1)	0.67
CXCL9	31.0 (32.1)	26.9 (23.4)	0.39
TNFRII	175.3(81.6)	169.8(83.5)	0.71
IL-6	12.3(7.8)	12.6(7.0)	0.82

*SD, standard deviation; N (%), number (percentage). P was calculated by the t-test or the Mann-Whitney U test or the Chisq test where appropriate. TNM stage, tumor-node-metastasis stage*.

### Association of Inflammatory Markers With DFS

**Figure 1:**
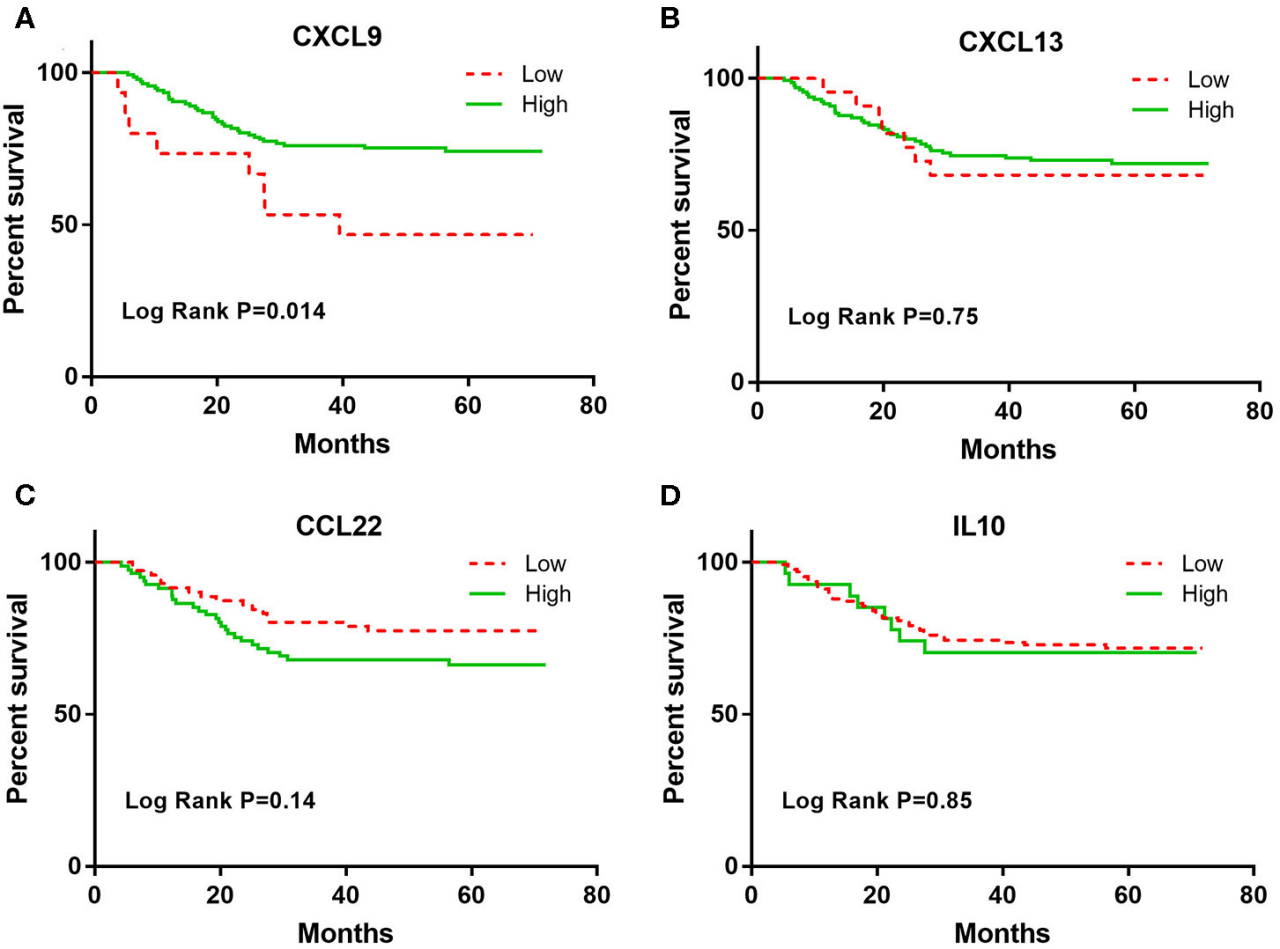
The Kaplan-Meier curves for the DFS among early stage lung adenocarcinoma patients according to CXCL9, CXCL13, CCL22, and IL-10 levels. **(A)** CXCL9, **(B)** CXCL13, **(C)** CCL22, and **(D)** IL10.

In the multivariate Cox analyses, only CXCL9 was significantly associated with DFS after adjusting for age, sex, smoking status, stage, and adjuvant therapy (Table 2). Lung adenocarcinoma patients with high CXCL9 levels had a 71% reduced risk of recurrence relative to patients with low CXCL9 levels (*HR* = 0.29, 95% CI: 0.13–0.64, *p* = 0.0021). K–M analysis showed that lung adenocarcinoma patients with high CXCL9 levels had a significantly longer median DFS time than those with low CXCL9 levels (log-rank test *p* = 0.014; Figure 1A). While other markers, such as CXCL13, CCL22, and IL-10, did not show significant differences in DFS of lung adenocarcinoma patients between high and low groups (Figures 1B–D). After Bonferroni correction, CXCL9 remained significant, with a significance level of 0.5% (0.05/10, here 10 refers to 10 biomarkers under study).

**Table 2:** Risk prediction of inflammation biomarkers for disease-free survival (DFS) of early stage lung adenocarcinoma.

**Biomarkers, pg/mL**	**Non-recurrence cases N (%)**	**Recurrence cases N (%)**	**HR (95% CI)**	P^*^
CXCL9				
≤ 7.8	7 (6.4)	8 (18.6)	1	0.0021
>7.8	102 (93.6)	35 (81.4)	0.29 (0.13–0.64)	
CXCL13				
≤ 21.4	15 (13.8)	7 (16.3)	1	0.33
>21.4	94 (86.2)	36 (83.7)	0.67 (0.30–1.52)	
CCL22				
≤ 77.7	55 (50.5)	16 (37.2)	1	0.27
>77.7	54 (49.5)	27 (62.8)	1.40 (0.76–2.56)	
TNFRII				
≤ 275.4	99 (90.8)	39 (90.7)	1	0.89
>275.4	10 (10.2)	4 (9.3)	1.10 (0.37–3.13)	
IL-6				
≤ 14.1	66 (60.6)	23 (53.5)	1	0.47
>14.1	43 (39.4)	20 (46.5)	0.80 (0.43–1.47)	
CRP				
≤ 888244.7	66 (60.6)	20 (46.5)	1	0.23
>888244.7	43 (39.4)	23 (53.5)	1.45 (0.79–2.65)	
IL-10				
Undetectable	90 (82.6)	35 (81.4)	1	0.40
Detectable	19 (17.4)	8 (18.6)	1.39 (0.64–3.03)	
IL-1b				
Undetectable	75 (68.8)	33 (76.7)	1	0.20
Detectable	34 (31.2)	10 (23.3)	1.65 (0.77–3.54)	
IFN-r				
Undetectable	67 (61.5)	26 (60.5)	1	0.30
Detectable	42 (38.5)	17 (39.5)	1.38 (0.15–2.56)	
TGF-a				
Undetectable	92 (84.4)	38 (88.4)	1	
Detectable	17 (15.6)	5 (11.6)	0.87 (0.34–2.22)	0.77

*HR, hazard ratio; 95% CI, 95% confidence interval*.

* *P was adjusted for age, gender, smoking, TNM stage, and adjuvant therapy under multivariate Cox proportional hazards model*.

**Figure 2:**
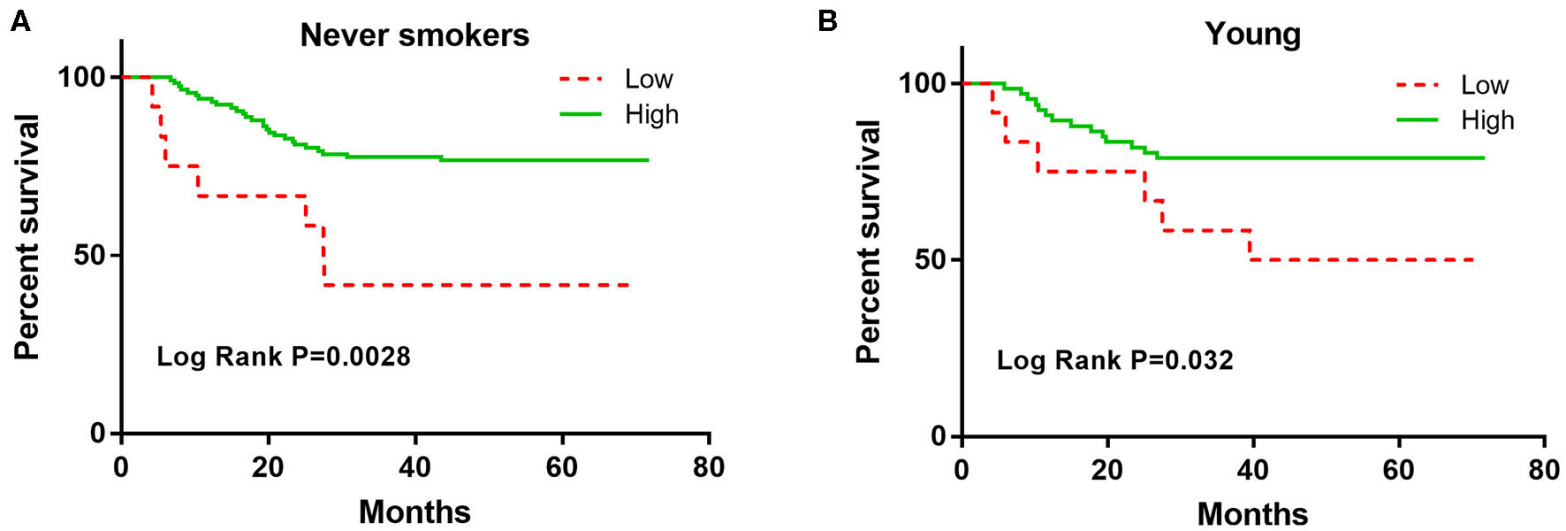
The Kaplan-Meier curves for the DFS among never smokers and young early stage lung adenocarcinoma patients according to CXCL9. **(A)** never smokers, **(B)** young.

The association of CXCL9 with DFS in lung adenocarcinoma patients was stratified by age, sex, and smoking status (Table 3). Elevated CXCL9 levels were found to predict a good prognosis in never smokers, females, and both young and old lung adenocarcinoma patients. The K-M analysis showed that increased CXCL9 levels were still significantly corelated with longer DFS in never smokers and young lung adenocarcinoma patients (Figures 2A,B).

**Table 3:** Stratified analyses of CXCL9 on disease-free survival in early stage lung adenocarcinoma.

**Variables**	**Non-recurrence cases N**	**Recurrence cases N**	**HR (95% CI)**	P^*^
Age <60				
Low/High	6/52	6/28	0.34 (0.13–0.91)	0.031
Age ≥ 60				
Low/High	1/50	2/42	0.08 (0.15–0.46)	4.3^*^10^−3^
Female				
Low/High	5/65	4/15	0.18 (0.05–0.62)	6.6^*^10^−3^
Male				
Low/High	2/37	4/20	0.33 (0.11–1.00)	0.051
Never smokers				
Low/High	5/89	7/27	0.10 (0.04–0.26)	3.0^*^10^−6^
Ever smokers				
Low/High	2/13	1/8	1.10 (0.09–13.60)	0.95

*HR, hazard ratio; 95% CI, 95% confidence interval*.

* *P was adjusted for age, gender, smoking, TNM stage, and adjuvant therapy under multivariate Cox proportional hazards model*.

### Influence of CXCL9 Expression on OS and TCGA Validation

Lung adenocarcinoma patients with high CXCL9 levels had an 80% reduced risk of death (*HR* = 0.20, 95% CI: 0.05–0.78, *p* = 0.021) relative to patients with low CXCL9 levels (Table 4), although K–M analysis did not reach statistical significance (log-rank test *p* = 0.082; Supplement Figure 1).

**Table 4:** Significant association of CXCL9 in overall survival (OS) of lung adenocarcinoma.

**Discovery**		**Validation**	
**HR (95% CI)**	***P***	**HR (95% CI)**	***P***
0.20 (0.05–0.78)	0.021	0.71 (0.45–0.99)	0.044

*(Shanghai Chest hospital, N = 152; TCGA, N = 492)*.

*HR, hazard ratio; 95% CI, 95% confidence interval*.

After analysis of the TCGA database, 492 lung adenocarcinoma patients were enrolled in the validation cohort, and their characteristics are summarized in Supplement Table 1. Patients with high CXCL9 levels in the validation cohort had a 29% reduced risk of death (*HR* = 0.71, 95% CI: 0.45–0.99, *p* = 0.044; Table 4).

## Discussion

To the best of our knowledge, this is the first study that evaluated multiple inflammatory biomarkers and prognosis for early-stage lung adenocarcinoma in a Chinese population. The key finding of the present study was that CXCL9, a chemokine, was associated with both the DFS and OS of early-stage lung adenocarcinoma patients.

CXCL9, a ligand of CXCR3, is secreted by various cell types, including immune cells (23–25) and non-immune cells (26, 27). CXCL9 plays a controversial role in tumor development and pathogenesis (28–30) and exhibits both positive and negative prognostic values for different tumor types (31). For example, Lieber et al. (32) found that ovarian carcinoma patients with high CXCL9 levels had significantly longer relapse-free survival (RFS) than patients with low CXCL9 levels. In contrast, Chang et al. (33) found that high serum CXCL9 levels predicted poor DFS and OS in oral squamous cell carcinoma (OSCC) patients. In a recent study (34), CXCL9 was also found to be related to the efficacy of PD-1 immunotherapy. Chow et al. (34) revealed the role of CXCL9 in recruiting intratumoral CXCR3^+^ CD8^+^ T cells and its use as a potential biomarker of the response to immunotherapy.

Regarding lung cancer, Addison et al. found high levels of CXCL9 in ninety NSCLC tissues and that CXCL9 could inhibit tumor-derived angiogenesis (35). However, a study conducted by Kowalczuk et al. (36) found that CXCL9 expression was low in NSCLC tumor tissues but not related to either DFS or OS. Moreover, Nakanishi et al. (37) indicated that CXCL9 was highly upregulated in tumor tissues, but no significant correlation between the CXCL9 level and DFS was observed. Bodelon et al. (38) investigated the correlation between 77 inflammatory markers and the prognosis of lung cancer, and CXCL9 levels were also not found to be related with long survival of NSCLC patients. In our previous study (12), a high CXCL9 level was found to be related to a reduced risk of lung adenocarcinoma. However, the association was non-significant after Bonferroni correction. In the present study, high CXCL9 levels were found to be related to prolonged DFS and OS in early-stage lung adenocarcinoma patients. Nevertheless, the underlying biological mechanisms of this correlation are required to better define the prognosis and likelihood of a therapeutic response in lung adenocarcinoma patients.

Our findings demonstrate for the first time that the CXCL9 level is as a protective factor for both the DFS and OS of early-stage lung adenocarcinoma patients. The application of CXCL9 as a predictive and prognostic biomarker of early-stage lung adenocarcinoma patients will be investigated in the future with large, well-designed, multicenter cohort studies, along with *in vitro* and *in vivo* functional experiments.

## Data Availability Statement

All datasets generated for this study are included in the article/Supplementary Material.

## Ethics Statement

This study was approved by the Ethics Committee of Shanghai Chest hospital, and all participants provided written informed consent.

## Author Contributions

BH, TC, and YZ contributed to the conception and design of the research. BS and MH contributed to the experiment. YZ, JQ, YL, and JL contributed to the analysis and interpretation of the data. XZ and HW contributed to the clinical data collections. YZ and JQ wrote the first draft of manuscript. All authors contributed to the article and approved the submitted version.

## Conflict of Interest

The authors declare that the research was conducted in the absence of any commercial or financial relationships that could be construed as a potential conflict of interest.

## Supplementary Material

Supplement Figure 1 The Kaplan-Meier curves for the OS among early stage lung adenocarcinoma patients according to CXCL9.

Supplement Table 1 Baseline characteristics of TCGA validation cohort patients.
