# Supplementary figures and images for: CXCL9 as a Prognostic Inflammatory Marker in Early-Stage Lung Adenocarcinoma Patients
Source: Front Oncol. 2020 Jun 30;10:1049. doi: 10.3389/fonc.2020.01049 (PMC7347039; doi:10.3389/fonc.2020.01049)

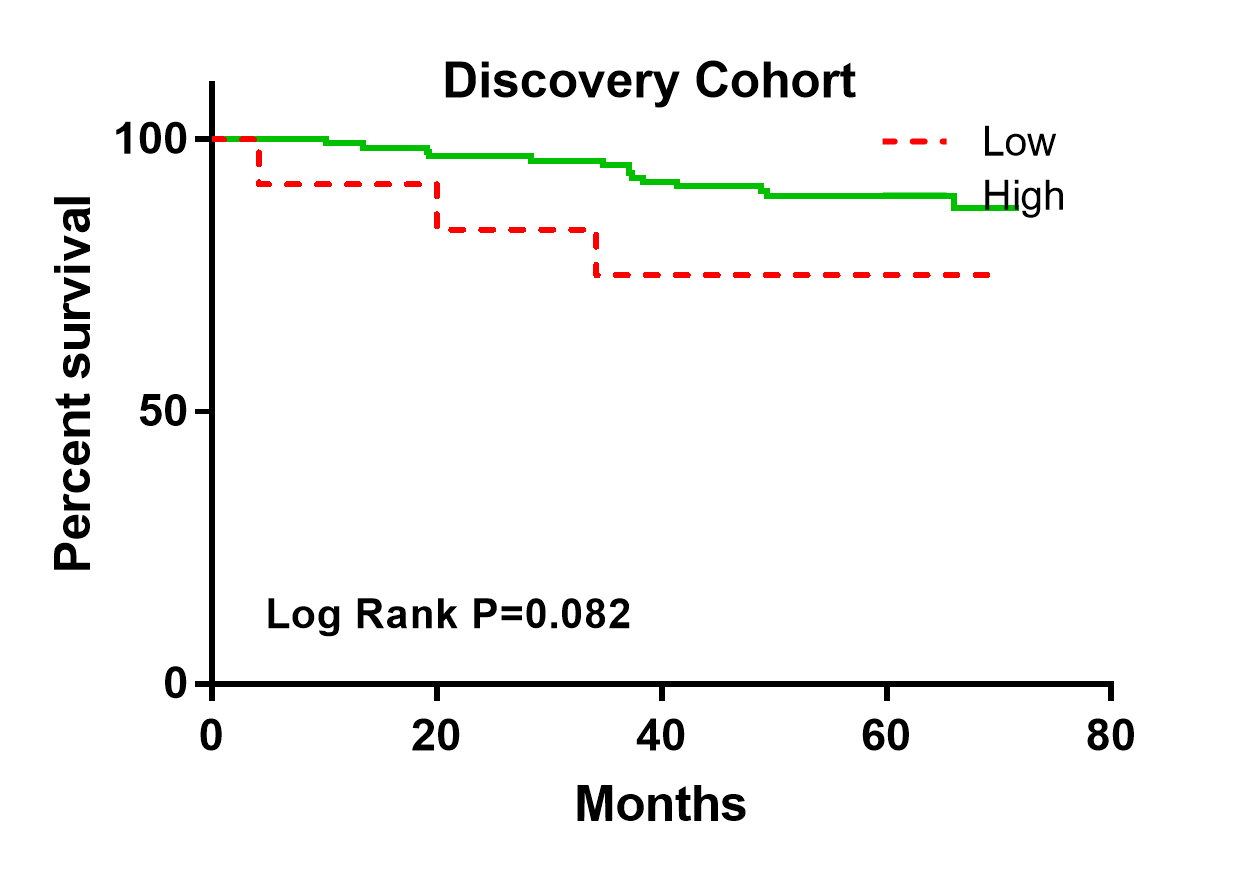

Supplement: Supplement Figure 1 — The Kaplan-Meier curves for the OS among early stage lung adenocarcinoma patients according to CXCL9. [file Image_1.TIF]
