# Supplementary material for: CXCL9 as a Prognostic Inflammatory Marker in Early-Stage Lung Adenocarcinoma Patients
Source: Front Oncol. 2020 Jun 30;10:1049. doi: 10.3389/fonc.2020.01049 (PMC7347039; doi:10.3389/fonc.2020.01049)
Supplement: Supplement Table 1 — Baseline characteristics of TCGA validation cohort patients. [file Table_1.DOC]

Supplement Table 1. Baseline characteristics of TCGA validation cohort patients

| **Variables** | **Number of patients（%）** |
| --- | --- |
| Age (years), mean (SD) | 64.6 (11.7) |
| Gender |  |
| Female | 227 (46.1) |
| Male | 265 (53.9) |
| Race |  |
| White | 298 (60.6) |
| Others | 194 (39.4) |
| Living Status |  |
| Dead | 180 (36.6) |
| Alive | 312 (63.4) |

*Abbreviations*: SD, standard deviation.
